# Sympathetic crashing acute pulmonary edema following pacemaker insertion

**DOI:** 10.1002/ccr3.7518

**Published:** 2023-06-09

**Authors:** Ibtehal Othman Hussain Mohammed, Sarya Swed, Weaam Ezzdean, Mohammad Badr Almoshantaf, Baraa Shebli, Bisher Sawaf, Abutalib Mohamed Ahmed Hamoda

**Affiliations:** ^1^ Faculty of Medicine University of Khartoum Khartoum Sudan; ^2^ Faculty of Medicine Aleppo University Aleppo Syria; ^3^ Department of Urology Ibn Al‐Nafees Hospital Damascus Syria; ^4^ Department of Neurosurgery Ibn Al‐Nafees Hospital Damascus Syria; ^5^ Cardiology Resident, Department of Cardiology Aleppo University Hospital, University of Aleppo Aleppo Syria; ^6^ Department of Internal Medicine Hamad Medical Corporation Doha Qatar; ^7^ Internal Medicine and Interventional Cardiologist and Fellowship Electrophysiology University of Alemam Almahadi Kosti Sudan

**Keywords:** acute kidney injury, hypertensive emergency, permanent pacemaker, pulmonary edema

## Abstract

**Key Clinical Message:**

Sympathetic crashing acute pulmonary edema (SCAPE) complicating pacemaker implantation is a very uncommon and dangerous occurrence. Following pacemaker implantation, patients need stringent monitoring, and compelling evidence about SCAPE treatment is required.

**Abstract:**

Sympathetic crashing acute pulmonary edema complicating a pacemaker insertion as the case in our patient is extremely rare. We report a case of 75‐year‐old man with a complete AV block, which requires urgent pacemaker implantation. Half an hour following the insertion of the pacemaker, an abrupt SCAPE emerged and the patient was incubated immediately.

## Graphical abstract

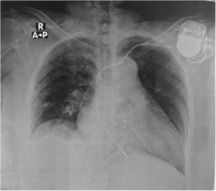

## 1 INTRODUCTION

Sympathetic crashing acute pulmonary edema (SCAPE) is the most severe form of acute pulmonary edema, an abrupt abnormal redistribution of fluid in the lungs, which is clinically diagnosed and treated immediately once it is diagnosed, as better outcomes is expected when managed appropriately.^1^

Patients with SCAPE usually have severe acute heart failure and present with abrupt onset of rapidly progression symptoms characterized by elevated cardiac filling pressures leading to acute accumulation of fluid in the pulmonary interstitial and alveolar spaces.^2^, ^3^

The diagnosis of SCAPE is purely clinical as most patients present with abrupt onset of shortness of breath, which progresses over minutes‐to‐hours.^4^, ^5^ Patients complain of severe respiratory distress and appear restless, diaphoretic, and hypoxic on arrival.^4^ Tachycardia and markedly elevated arterial pressure are considered a hallmark that suggest elevated sympathetic activity.^5^

Cardiac sensitive troponin is markedly elevated in significant proportion of patients with heart failure and is associated with increased mortality.^6^ Moreover, screening echocardiography during the acute presentation would reveal preserved ejection fraction >0.5, which points to worsening of diastolic dysfunction as the underlying mechanism of this severe subtype of acute pulmonary edema.^7^, ^8^

Patients with congestive heart failure has higher risk of developing serious cardiac arrhythmias which also increases the mortality rate and make them prone to decompensation, which could cause acute pulmonary edema.^9^ Multiple factors contribute to arrhythmias in congestive heart failure such as, myocardial ischemia, catecholamines, left ventricular dysfunction, electrolyte disturbances, and drugs used to treat heart failure, management of CHF patients should be targeted to reduce and correct these influences in order to prevent cardiac arrhythmias in those patients.^9^

Cardiac arrhythmias might lead to cardiac decompensation, which could be life‐threatening event if sever acute pulmonary edema is developed. Thus, treatment of these arrhythmias is mandatory, and should be initiated immediately once it is diagnosed.^10^

A variety of management have been described for cardiac arrhythmias in the medical literature and pacemaker implantation is considered one of these treatment when indicated,^11^ as it is considered an extremely rare case for pacemaker implantation to cause acute cardiac decompensation provoking a severe form of acute pulmonary edema, which is SCAPE, that we are reporting in the following case.

## 2 CASE PRESENTATION

A 75‐year‐old male presented to the cardiology center complaining of new‐onset dyspnea and lower limbs swelling associated with dizziness and syncopal attacks since 3 h ago. The patient has a medical history of hypertension for 11 years, gouty arthritis. He takes regularly (candesartan 16 mg, amlodipine 10 mg, allopurinol 100 mg); he has otherwise no significant medical, surgical or allergic history. The patient is neither a smoker nor alcoholic.

Upon clinical examination of the cardiovascular system, the heart sounds were normal without any associated mummer, S4 sound was heard, blood pressure measured 140/80 mmHg, heart rate was 49 bpm. Regarding the respiratory system, the chest was clear upon auscultation, SpO_2_ was 98% in room air, and reparatory rate was 16 rpm. Clinical examination of the limbs revealed severe pitting edema over the tibia. Further examination of other organ systems did not reveal any significant abnormality.

Subsequent electrocardiogram (ECG) revealed complete heart block (third degree AV block), and the patient was selected for an elective permanent pacemaker (VVIR) implantation. An echocardiography showed normal left ventricular systolic function (ejection fraction = 60%) with Grade 1 left ventricular diastolic dysfunction, mild dilation in most heart chambers (except for the left ventricle which is normal), mild left ventricular hypertrophy, moderate mitral regurgitation, and moderate pulmonary hypertension. Laboratory test results prior to the operation were: HGB: 12.8 g/dL, WBC: 11.05 × 10^3^/μL, PLT: 285 × 10^3^/μL, urea: 134 mg/dL, Cr: 2.9 mg/dL, K^+^: 3.1 mmol/L, Na^+^: 144 mmol/L, INR: 1.2.

The procedure went smoothly and the patient was transferred safely to the recovery room. Sudden severe dyspnea emerged half an hour following the procedure. Upon physical examination, fine crackles were heard all over the chest, SpO_2_ was 60% in room air, and blood pressure measured 200/120 mmHg, paced heart rate was 60 bpm. The patient was transported immediately to the cardiac care unit and was intubated and put on mechanical ventilation with the ventilation set on synchronized intermittent mandatory ventilation (SIMV) mode. The patient was put immediately on nitroglycerine infusion pump starting with a dose of 3 mg per hour and then modified according to vital signs to overcome the overwhelming pulmonary edema. He was also managed with 40 mg of furosemide and Meropenim (given for the increased WBCs count).

**FIGURE 1:**
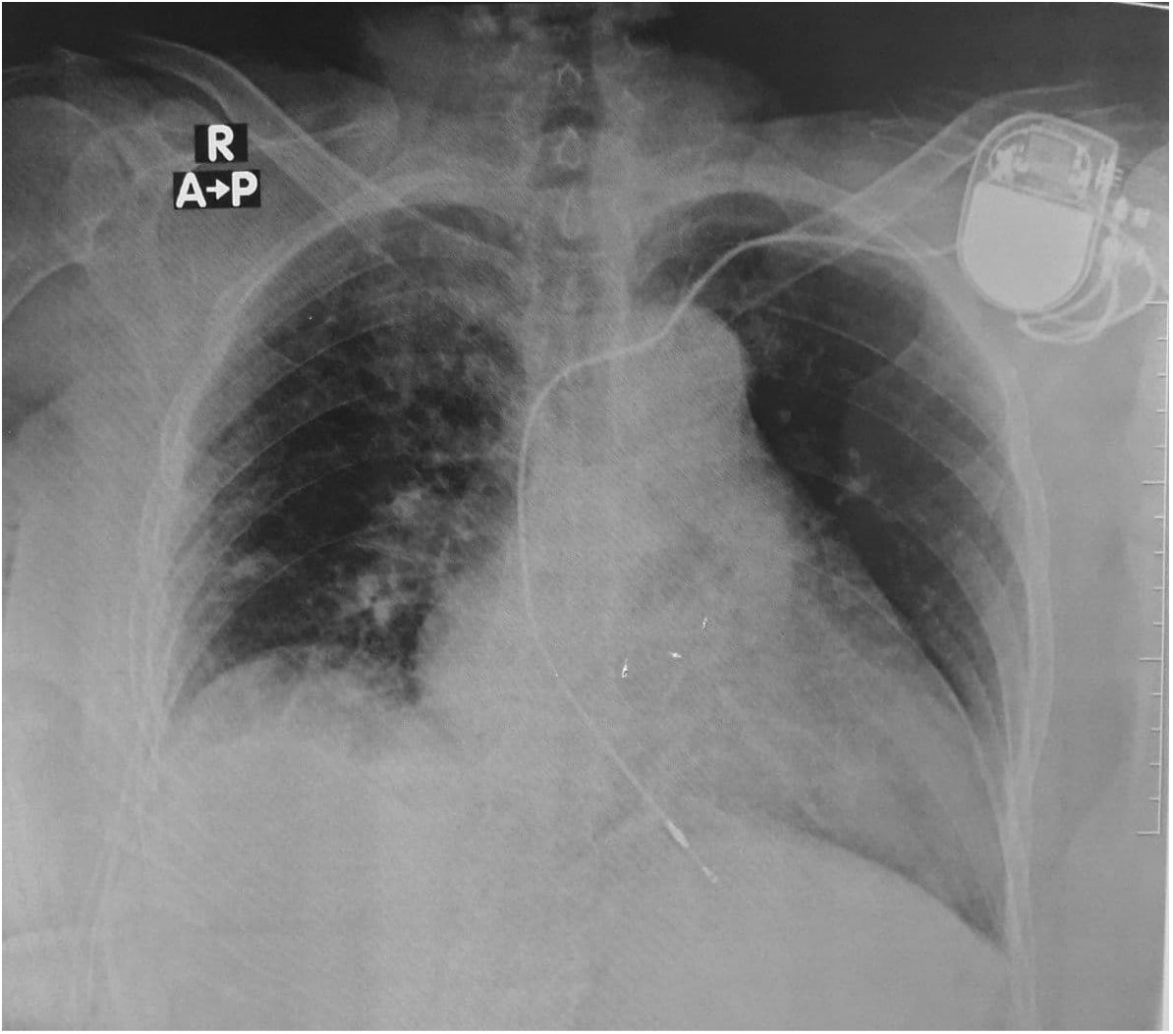
A CXR image of the patient after insertion of the pacemaker.

A Chest X‐Ray (CXR) showed pulmonary hilar congestion with pleural effusion (Figure 1). Arterial blood gas (ABG) analysis of the patient showed the following results: (PH: 7.41, PCO_2_: 34.7 mmHg, PO_2_: 50.4 mmHg, HCO_3_: 22 m Eq/L). The patient was monitored closely; SpO_2_ measurements were between 97% and 100%; Blood pressure measurements dropped soon afterwards and ranged between 150/60 and 180/95 the following 2 days, troponin test was negative; other labs did not show any significant change and WBCs were still increased. After 72 h, vital signs stabilized and the patient was extubated uneventfully. Two days later, an abdominal ultrasound showed signs of chronic kidney disease (CKD) which is the probable cause of elevated kidney function tests; a significant pleural effusion was also detected during the ultrasound, and a right inguinal hernia was also detected. Afterwards, the patient was discharged successfully with a blood pressure of 140/80 mmHg and SpO_2_ of 98% in room air. He was prescribed furosemide 40 mg QD, spironolactone 25 mg QD, valsartan 160 mg QD, amlodipine 10 mg QD, cefpodoxime 200 mg and ciprofloxacin 500 mg.

At 3 months follow‐up, we noticed acceptable improvement of the patient situation with no accompanying complications.

## 3 DISCUSSION

We believe that it is clinically difficult to differentiate SCAPE from acute heart failure symptoms (AHFS) as their symptoms may intersect.^1^ SCAPE is the most extreme type of the APE spectrum. It develops quickly with increasing afterload resulting in a decompensating patient. Thus, quick intervention with high‐dose of nitroglycerin has been widely used to treat SCAPE. As nitroglycerin helps to reduce both preload and afterload, concerns of severe hypotension were raised.^12^ Lack of the universally accepted diagnostic criteria of SCAPE, and controversy in treatment methods, are complicating the treatment process.

Nitroglycerin is being used for both SCAPE and AHFS. AHFS require doses of 10–20 μg/min to decrease preload. On the other hand, using nitroglycerin in SCAPE is mainly targeting the afterload with much higher doses.^13^ Yet, there are still many studies which are suggesting different doses and approaches for the use of nitroglycerin in SCAPE.^14^, ^15^

In our case, high doses of nitroglycerin (starting at 50 μg/min) were given through infusion pump that resulted in symptoms relief. Furosemide was also given as a common standard treatment for APE spectrum. Despite the above, no signs of hypotension were documented throughout the admission. Similarly, Stemple et al. have described case series of four cases of SCARE that were all managed with high doses of nitroglycerin infusions. All four patients achieved symptomatic improvement and required no additional acute pharmacologic therapy or invasive mechanical ventilation.^16^

The main target for our case is the put the spot light on the idea of SCAPE potentially complicating pacemaker insertion. We extensively searched the literature to find similar cases. We used PubMed, Google scholar, and EMBASE. We only found one case of the incidence of flash pulmonary edema (another name for SCAPE) after pacemaker insertion.^17^ Párraga et al., believe that placement of the pacemaker lowered the heart rate which generated decrease in heart output. This allowed the triggering of the renin angiotensin system and resulted in increased blood pressure, allowing for secondary pulmonary congestion and SCAPE to develop. Despite the justified logic behind this theory, it's much early to locate the true reason for SCAPE to occur after pacemaker insertion due to the small numbers of reported cases at the moment. Underlying pathologies such as renal artery stenosis and atheromatous vascular diseases, may play a role in SCAPE.

SCAPE is clinical entity that is yet being completely understood in the literature. Our case's clinical presentation was unusual with unpredicted complication arising. Pacemaker insertion is known to have several common complications but SCAPE was only reported once before. We emphasize that clinical suspicion should be taken when similar symptoms to our case appear after pacemaker insertion. Additional caution is advised toward the elderly as we doubt that they should be more susceptible to SCAPE due to their generally poor compensating mechanisms. For now, nitroglycerin should be considered the first line management for SCAPE until more verified studies take place. Diagnosis timing is vital and there should be no delay in recognizing and managing SCAPE. This can be achieved by clinically observing pulmonary congestion signs in patients with postplacement of pacemaker.

## 4 CONCLUSION

SCAPE is an uncommon clinical condition with a poorly known etiology. SCAPE following a pacemaker implantation, as in our patient's case, is exceedingly rare; therefore, it highlights the need of patient strict monitoring following pacemaker insertion and the need for robust data about SCAPE therapy.

## AUTHOR CONTRIBUTIONS

**Ibtehal Othman Hussain Mohammed:** Conceptualization; writing – original draft; writing – review and editing. **Sarya Swed:** Writing – original draft; writing – review and editing. **Weaam Ezzdean:** Writing – original draft; writing – review and editing. **Mohammad Badr Almoshantaf:** Writing – original draft; writing – review and editing. **Baraa Shebli:** Writing – original draft; writing – review and editing. **Bisher Sawaf:** Writing – review and editing. **Abutalib Mohamed Ahmed Hamoda:** Writing – review and editing.

## FUNDING INFORMATION

This research did not receive any specific grant from funding agencies in the public, commercial, or not‐for‐profit sectors.

## CONFLICT OF INTEREST STATEMENT

All authors declared no conflict of interest.

## ETHICS STATEMENT

This case report did not require review by Ethics committee, University of Alemam Almahadi, Sudan.

## CONSENT

Written informed consent was obtained from the patient for publication of this case report and accompanying images. A copy of the written consent is available for review by the Editor‐in‐Chief of this journal.

## Data Availability

All data generated or analyzed are included in this article.

## References

[1] Agrawal N , Kumar A , Aggarwal P , Jamshed N . Sympathetic crashing acute pulmonary edema. Indian J Crit Care Med. 2016;20(12):719‐723. doi:10.4103/0972-5229.195710 28149030 PMC5225773

[2] Howlett JG . Current treatment options for early management in acute decompensated heart failure. Can J Cardiol. 2008;24:9B‐14B. doi:10.1016/S0828-282X(08)71023-7 PMC2794440 18629382

[3] Fromm RE , Varon J , Gibbs LR . Congestive heart failure and pulmonary edema for the emergency physician. J Emerg Med. 1995;13(1):71‐87. doi:10.1016/0736-4679(94)00125-1 7782629

[4] Kramer K , Kirkman P , Kitzman D , Little WC . Flash pulmonary edema: association with hypertension and reoccurrence despite coronary revascularization. Am Heart J. 2000;140(3):451‐455. doi:10.1067/mhj.2000.108828 10966547

[5] ‐ Gandhi SK , Powers JC , Nomeir AM , et al. The pathogenesis of acute pulmonary edema associated with hypertension. N Engl J Med. 2001;344(1):17‐22. doi:10.1056/NEJM200101043440103 11136955

[6] ‐ Perna ER , Macín SM , Parras JI , et al. Cardiac troponin T levels are associated with poor short‐ and long‐term prognosis in patients with acute cardiogenic pulmonary edema. Am Heart J. 2002;143(5):814‐820. doi:10.1067/mhj.2002.120152 12040342

[7] Obokata M , Reddy YNV , Borlaug BA . Diastolic dysfunction and heart failure with preserved ejection fraction: understanding mechanisms by using noninvasive methods. JACC Cardiovasc Imaging. 2020;13(1 Pt 2):245‐257. doi:10.1016/j.jcmg.2018.12.034 31202759 PMC6899218

[8] Kapila R , Mahajan RP . Diastolic dysfunction. Anaesth Crit Care Pain. 2009;9(1):29‐33. doi:10.1093/bjaceaccp/mkn046

[9] Parmley WW . Factors causing arrhythmias in chronic congestive heart failure. Am Heart J. 1987;114(5):1267‐1272. doi:10.1016/0002-8703(87)90215-8 3314442

[10] Greene HL . Clinical significance and management of arrhythmias in the heart failure patient. Clin Cardiol. 1992;15(Suppl 1):I13‐I21. 1395210

[11] Bax JJ , Marwick TH , Molhoek SG , et al. Left ventricular dyssynchrony predicts benefit of cardiac resynchronization therapy in patients with end‐stage heart failure before pacemaker implantation. Am J Cardiol. 2003;92(10):1238‐1240. doi:10.1016/j.amjcard.2003.06.016 14609610

[12] Paone S , Clarkson L , Sin B , Punnapuzha S . Recognition of sympathetic crashing acute pulmonary edema (SCAPE) and use of high‐dose nitroglycerin infusion. Am J Emerg Med. 2018;36(8):1526.e5‐1526.e7. doi:10.1016/J.AJEM.2018.05.013 29776826

[13] EMCrit 1 – Sympathetic Crashing Acute Pulmonary Edema (SCAPE). Retrieved February 4, 2022. https://emcrit.org/emcrit/scape/

[14] Wilson SS , Kwiatkowski GM , Millis SR , Purakal JD , Mahajan AP , Levy PD . Use of nitroglycerin by bolus prevents intensive care unit admission in patients with acute hypertensive heart failure. Am J Emerg Med. 2017;35(1):126‐131. doi:10.1016/J.AJEM.2016.10.038 27825693

[15] Levy P , Compton S , Welch R , et al. Treatment of severe decompensated heart failure with high‐dose intravenous nitroglycerin: a feasibility and outcome analysis. Ann Emerg Med. 2007;50(2):144‐152. doi:10.1016/J.ANNEMERGMED.2007.02.022 17509731

[16] Stemple K , DeWitt KM , Porter BA , Sheeser M , Blohm E , Bisanzo M . High‐dose nitroglycerin infusion for the management of sympathetic crashing acute pulmonary edema (SCAPE): a case series. Am J Emerg Med. 2021;44:262‐266. doi:10.1016/J.AJEM.2020.03.062 32278569

[17] Reinoso Párraga P , Sáez Nieto C , Ponce Dorrego MD , Murillo Gayo C . Flash pulmonary edema in a geriatric patient after the placement of a pacemaker: clinical case and literature review. Rev Esp Geriatr Gerontol. 2021;56(5):316‐319. doi:10.1016/J.REGG.2021.02.010 33775431

